# A Survey of Chinese Citizens’ Perceptions on Farm Animal Welfare

**DOI:** 10.1371/journal.pone.0109177

**Published:** 2014-10-14

**Authors:** Xiaolin You, Yibo Li, Min Zhang, Huoqi Yan, Ruqian Zhao

**Affiliations:** 1 History of Science, Nanjing Agricultural University, Nanjing, P.R. China; 2 Department of Sociology, Nanjing Agricultural University, Nanjing, P.R. China; 3 Department of Laws, Nanjing Agricultural University, Nanjing, P.R. China; 4 Philosophy of Science and Technology, Nanjing Agricultural University, Nanjing, P.R. China; 5 Key Laboratory of Animal Physiology and Biochemistry, Nanjing Agricultural University, Nanjing, P.R. China; Université Pierre et Marie Curie, France

## Abstract

Farm animal welfare has been gradually recognized as an important issue in most parts of the world. In China, domestic animals were traditionally raised in backyard and treated as an important component of family wealth. Industrialization of animal production brings forth the farm animal welfare concerns recently in China, yet the modern concept of animal welfare has not been publicized and a comprehensive recognition on how consumers and farmers perceive animal welfare is lacking. Therefore, we conducted a survey on public opinions toward farm animal welfare in China, based on pigs (including sows, piglets, and fattening pigs), domestic fowls (including layers and broilers) and their products. From 6,006 effective questionnaires approximately two thirds of the respondents had never heard of ‘animal welfare’; 72.9% of the respondents claimed that, for the sake of animal derived food safety, human beings should improve the rearing conditions for pigs and domestic fowls; 65.8% of the respondents totally or partly agreed on establishing laws to improve animal welfare; more than half of the respondents were willing, or to some extent willing, to pay more for high-welfare animal products, whereas 45.5% of the respondents were not willing or reluctant to pay more. In summary, farm animal welfare is still in its early stage of development and more efforts are needed to improve the public conception to animal welfare in the process of establishing farm animal welfare standards and legislations in China.

## Introduction

Concerns for the well-being of animals have long been attached importance in history of human beings. Nowadays, over 100 countries have enacted variety of laws on animal welfare, which not only demonstrates people’s respect for animals but also guarantees the safety of animal derived food. Farm animal welfare is also crucial in issues such as international trade, human health, and the environment protection. In China, animal well-being concerns can be traced back to ancient times in some literatures, such as “kindness to humans and other creatures” and “loving human and every creature”, which have become prevalent quotations among Chinese people for generations.

Recently, researchers carried on surveys on societal attitudes to animal welfare. A working paper of “Animal Welfare Project” presents a description of some major findings from surveys carried out in seven countries (France, the United Kingdom, Hungary, Italy, the Netherlands, Norway, and Sweden) in September 2005. The paper concentrated on describing national variations in public views on farm animal welfare in general and related shopping practices. The analyses indicate some common features in public opinions about farm animal welfare across Europe, and draw a conclusion that farm animal welfare is clearly an important issue for ordinary people across Europe. It is also found that, even though to a more limited extent, many Europeans still think about animal friendliness when shopping for food and making responsible purchases depends on what people mean by animal friendliness [1].

In a survey on United States households, researchers found sharp differences between direct and indirect questions related to farm animal welfare. This finding, coupled with the extant literature on indirect questioning, suggests that people’s concerns for farm animal welfare are actually much lower than what they say they are. It is suggested that responses from indirect questions provide a very different picture of the importance of farm animal welfare to the public than what might be suggested both by direct questioning [2].

In Belgium, a research has been conducted to develop a conception of farm animal welfare that starts from the public’s perception and integrates the opinion of different stakeholder representatives. The resulting conception revealed seven dimensions grouped in two different levels. Three dimensions were animal-based: “Suffering and Stress’’, “Ability to Engage in Natural Behavior’’ and “Animal Health’’. Four dimensions were resource-based: “Housing and Barn climate’’, “Transport and Slaughter’’, “Feed and Water’’ and ‘‘Human-Animal Relationship’’ [3].

Opinion surveys indicate that concerns about animal welfare resonate with the general public. In 2005, the European Commission’s Health and Consumer Protection Directorate General commissioned a comprehensive survey of public attitudes towards animal welfare, involving 24,708 citizens in 25 Member States of the European Union. Only 32% of respondents had a positive view about the welfare of laying hens and 22% had a very negative view of their welfare. More than 40% of respondents chose laying hens and broilers among the top three species needing improvements in their welfare. However, there are regional differences in the level of concern for animal welfare, and only 52% of respondents reported that they consider animal welfare when they are making their food purchases. Similarly, in an American Farm Bureau sponsored survey, more than 60% of respondents felt that the government should take an active role in promoting farm animal welfare, and 69–88% of respondents agreed with the statement “I would vote for a law in my state that would require farmers to treat their animals more humanely”. Fifty-six percent of respondents in this study felt that decisions about animal welfare should be made by the “experts” rather than the public. Interestingly, a survey of animal science faculty at US universities revealed support for general principles of animal welfare, and greatest concerns were directed at the welfare of poultry relative to other food producing species [4].

However, in China, animal welfare is still at the early stage of development. It didn’t draw attention from Chinese public until 2003. In effect, there is a long way to go for China to promote animal welfare. As China has its distinctive history and mode of economic and social development, it is necessary to investigate the public attitude towards animal welfare before establishing strategies of how to promote it more efficiently. This survey, as a part of the “Project of Research and Demonstration on Key Technological System for Farm Animals and Fowls”, is conducted to investigate the societal attitudes of the Chinese public towards animal welfare.

## Methods

### Problem and Strategy

Currently, the subjects of welfare of animals are diverse, including farm animals, experiment animals, and working animals, etc. This study exclusively focuses on the public attitudes to farm animals, with pigs and domestic fowls selected as question topics in that the scope of study can be narrowed and the reality and tradition of China be considered. In modern farming system of China, pigs and domestic fowls and their products are two main sources of meat consumption. They are the two animals of the ‘Six Farm Animals’ described in Chinese historical literatures. Therefore, the study on animals with which the public are most familiar and keep most relations will probably be the most appropriate initiation.

In accordance with the general interpretation of scholars worldwide, the scope of animal welfare covers the following five aspects that came to be known as the Five Freedoms, proposed by Farm Animal Welfare Council (now the Farm Animal Welfare Committee) and were pivotal in the advancement of animal welfare worldwide:

1. Freedom from thirst, hunger or malnutrition by ready access to fresh water and a diet to maintain full health and vigor.
2. Freedom from discomfort by providing a suitable environment including shelter and a comfortable resting area.
3. Freedom from pain, injury, and disease by prevention or rapid diagnosis and treatment.
4. Freedom to express normal behavior by providing sufficient space, proper facilities, and company of the animal’s own kind.
5. Freedom from fear and distress by ensuring conditions and treatment which avoid mental suffering [5].

This comprehensive survey, based on the 5 aspects mentioned above, designs a series of questions to get answers from respondents to know the public attitudes to animal welfare. The backgrounds of respondents such as gender, age, level of education, career, income, birthplace, and working place are considered as independent variables based on the hypothesis that these variables might have impact on the China’s public attitudes to animal welfare. Their relations to public attitudes are analyzed through Bi-category Logistic Model.

### Data

The data used in this study are collected from a questionnaire survey in January 2011 when the researchers allocated the questionnaires to a number of undergraduate students who brought them back to their hometowns to do the investigation. The survey was planned to cover every provinces and autonomous regions in China, with 4–5 cities or counties selected from each of them. For each city or county, 50 questionnaires have been distributed. Adding up, there have been totally about 8,000 questionnaires collected from all the areas surveyed. However, due to the restriction of admission quota of the researcher’s university, few students from Tibet, Hainan, Taiwan, Hong Kong, and Macao are available for this survey. (Nanjing Agricultural University annually admits only 4 undergraduate students from Tibet and only about 10 from Hainan province). With the exception of those five areas, this survey covers the remaining 29 provinces in China.

Before the survey, all students to do the survey got through necessary training, especially the ethical requirements of conducting survey faithfully. In the questionnaire, the item ‘Telephone Number of Respondent’ is designed to verify the effectiveness of the survey to ensure that every questionnaire has been completed properly. Finally, 6,006 effective questionnaires were received, accounting for 75.1% of total questionnaires.

During the survey, the students are allowed to use non-random approach to select respondents with the gender ratio being kept as approximately 1∶1. In all respondents of this survey, the male interviewees account for 51.5%, and female ones 48.5%, which approximates the gender ratio of China. As for the age proportions of samples, 17.3% are below 20 years old; 44.9% between 21 and 34 years old; 27.6% between 35 to 49 years old; 7.0% between 50 to 59 years old; and 2.9% above 60 years old. With regard to the level of education, 5.5% are below the level of elementary school; 15.1% are at the level of junior high school; 24.5% reach the level of senior high school; and 47.0% receive higher education. These data show that the respondents reflect relatively low age and high education level, not precisely representing the real percentages of China. The reason probably lies in the fact that the students conducting the survey most likely to send questionnaires to their peers. At least, the survey results can reflect a trend on public perception to this issue.

### Variables

#### (1) Variable Declaration and Value Assignment

The main focus of this survey will be put on four issues: first, public awareness of the concept and connotation of ‘animal welfare’; second, public opinions on current intensive factory rearing; third, the public’s level of satisfaction on legislation of animal welfare; fourth, the public’s level of contentment on the market supply of pork and egg. Practical questions have been designed for each category and all variables are bi-category variables expressed as Vi(i = 1, 2, ……, 9) as in Table 1.

**Table 1:** Variables and Assignment.

**Independent variables**	Gender	male = 1, female = 2
	Age	below 34 = 1, 35–59 = 2, 60 = 3
	Education	below elementary = 1, junior high = 2, senior high = 3, college and above = 4
	Career category	farmer = 1, urban and rural non-agriculture employee = 2, professional = 3, government and NGO employee = 4
	Annual household income	below 40,000 Yuan = 1, 40,000–80,000 = 2, 80,000–150,000 = 3, above 150,000 = 4
	Birthplace (urban or rural)	urban = 1, rural = 2
	Birthplace (eastern,central or western)	eastern = 1, central = 2, western = 3
	Working place (urban or rural)	urban = 1, rural = 2
	Working place (eastern, central or western)	eastern = 1, central = 2, western = 3
**Dependent variables**	Awareness ofconcept and connotation ofanimal welfare	
	V1 being aware ofanimal welfare or not	Y = 1, N = 0
	V2 being appropriate or not to use cementfloor for raising pig	Y = 1, N = 0
	V3 being appropriateor not to kill fowls nearcages in which they are kept	Y = 1, N = 0
	Attitude and response tofactory rearing	
	V4 evaluation on factory rearing	positive = 1, negative = 0
	V5 willing or notwilling to pay morefor meat products for thesake of animal welfare	Y = 1, N = 0
	Legislative issues onanimal welfare	
	V6 agreeing or not agreeing on establishinglegislation for animal welfare	Y = 1, N = 0
	V7 agreeing or not agreeing on introducing foreignlegislations of animal welfare into China	Y = 1, N = 0
	The satisfaction degree of public to pork and egg supply	
	V8 satisfaction degreeon pork supply	satisfy = 1, dissatisfy = 0
	V9 satisfaction degreeon egg supply	satisfy = 1, dissatisfy = 0

In this study, the elements of gender, age, education, career, income, and locality have been designed as independent variables, some of which are partly adjusted in order to be more conveniently analyzed by Logistic Model. The scope of age variables has been narrowed down from original 5 levels to 3 levels of ‘youth, middle age, and senior’. The scope of income variables has been reduced from original 6 levels to 4 levels, with the family income of below 40,000 Yuan merged into only one level. The career variables, initially defined as constant category variables, have been divided into 4 levels based on the technical characteristics of industry or career attributes as well as its social power. The two variables of birthplace and working place initially had 9 levels, but in analysis they have sub-variables of ‘urban or rural’ and ‘eastern, central or western’. Table 1 shows the assignment on independent variables.

#### (2) The Mutual Impacts between Independent Variables and Verification

There may exit some mutual impacts between independent variables. Hence, it is critical to measure such impacts. Table 2 shows the Kendall's tau-b coefficient results between independent variables.

**Table 2:** Kendall’s tau-b Coefficient Results of Independent Variable Pairs.

	Gender	Age	Birthplace	Education	Workingplace	Career
Age	−0.055**					
Birthplace	–0.013	0.027*				
Education	0.027	–0.418**	–0.147**			
Workingplace	–0.029*	0.182**	0.736**	–0.284**		
Career	0.033*	–0.155**	–0.054**	0.227**	–0.126**	
Income	–0.024	–0.068**	–0.235**	0.262**	–0.195**	0.027

Note: **P<0.01 and *P<0.05 were through dual-trail verification.

In Table 2, there are 17 corelativities among all 21 independent variable pairs. In the dual-trail verification on condition of P<0.01, the corelativity between ‘birthplace’ and ‘working place’ demonstrates the maximum of ‘0.736’, indicating that there is fairly large overlap between the place of birth and work. The second maximum is −0.418 between ‘age’ and ‘education’, implying that the lower the age is, the higher education will be received. Other corelativities are all less than |0.3|. Although Table 2 reflects the corelativities between independent variable pairs, the massive number of 6006 samples itself can overcome the extreme correlativity (for instance, r>0.95) between variable pairs and thus achieve a high standard capacity of statistics. This advantage can avoid any interpretation problem caused by multicollinearity between independent variable pairs. Therefore, the bi-category Logistic regression model can be utilized to analyze and verify the significance of model fitting variation.

This study has been approved by Ethics Committee of Scientific Research of Nanjing Agricultural University, and the respondent information is anonymized.

## A Descriptive Analysis of the Public Attitudes to ‘Animal Welfare’

### The Public Cognition of Concept and Connotation of Animal Welfare

As revealed in the survey with 5,982 respondents, 2,187 of them (36.6%), a little more than one third, has ever heard of ‘animal welfare’. In other words, the majority of the public did not ever hear of this concept. But it’s undeniable that ideas of treating animals with love, which can be found in Chinese traditional culture, are similar to the concept of animal welfare in the western culture. Such ideas include “kindness to humans and other creatures” and “loving human and every creature”. To measure China’s public awareness about animal welfare, two sets of questions have been designed by the researchers.

The first set of questions includes three statements with focus shifting from human beings to animals. The first statement is human-centered: “Pigs and domestic fowls are only beast, and people can treat them as they wish”. The second one sees animals as tools: “Humans should improve the rearing conditions for pigs and domestic fowls to ensure the quality and safety of animal products”. The third one says that animals should have some basic rights: “Pigs and domestic fowls should enjoy happy life and be free from troubles as humans do”. The results show that, among 5,916 respondents, 4,314 of them (72.9%) choose an “instrumental reason” to decide how humans should treat animals; 1,135 of them (19.2%) agree that animals themselves should enjoy some basic rights; and 468 people (7.9%) support anthropocentrism. So it can be inferred that the majority of Chinese public treat animals as instruments and part of the public think that animals themselves should enjoy some basic rights, the number of whom is 1.43 times larger than those who assert that “Pigs and domestic fowls are only beast, and people can treat them as they wish”.

The second set of questions involves two common situations about pigs and fowls in the daily lives of Chinese people. The public attitude is sought by investigating the public opinions on these matters. Table 3 shows the statistical results as follows.

**Table 3:** China’s Public Attitudes to the Way of Treating Pigs and Domestic Fowls in their Daily Life.

	Though pigs naturallylike to nose the earth, mostof the piggeries use cement floor.		The venders killfowls near the cages in which fowls are kept.	
	n	%	n	%
Appropriate	886	15.0	618	10.4
Somewhat inappropriate	2913	49.2	2581	43.5
Extremely inappropriate	1215	20.5	1827	30.8
Unimportant	911	15.4	900	15.2
N	5925	100.0	5926	100.0

Table 3 emphasizes two points: first, behavioral welfare to give animals freedom to live in a natural way; second, psychological welfare to avoid anxiety and fear. As shown in the data about animal behavioral welfare (take pig rearing as an example), 20.5% of respondents think it is “extremely inappropriate” to rear pigs on cement floor, 49.2% of respondents choose “somewhat inappropriate”, these two groups totaling 69.7%. However, 15.0% consider this acceptable and 15.4% don’t care. In the meantime, as for animal psychological welfare by the example of fowl killing, 30.8% of respondents consider killing fowls near cage as “extremely inappropriate” and 43.5% view it as “somewhat inappropriate”, with a total percentage of 74.3%. Only 10.4% support this and 15.2% choose “unimportant”. The result reveals that most of the answers correspond to the ideas of animal welfare.

### A Description of Public Attitudes to Factory Rearing

To find out public attitude to factory rearing in China, four choices are given for respondents to select: factory rearing is “a very good way of production”, “a scientific way of production”, “a way limiting the freedom of pigs and domestic fowls”, or “a cruel way of production”. Among the 5,705 respondents, 1,228 of them (21.5%) select “a very good way of production”; 1,970 of them (34.5%) believe it is a scientific way; 1,357 people (23.8%) think this way limits the freedom of pigs and domestic fowls; 1,150 respondents (20.2%) dismiss this as a cruel way. The above data show serious discrepancies among people’s evaluations of the current mode of factory farming in China: a little more than half (56%) show positive attitude and a little less than half (44%) express negative opinion.

When giving comprehensive evaluation of current factory rearing modes, the respondents are also asked to further evaluate the details of factory rearing. The findings are listed in Table 4.

**Table 4:** China’s Public Attitudes to the Process and Final Products of Factory Rearing of Pigs and Domestic Fowls (Multiple Choices Can Be Made).

	Number of people who approve	Number of effective sample	Proportion		Number of people who approve	Number of effectivesample	Proportion
Bad taste	2894	5987	48.3	high productivity	1991	5986	33.3
Overusing additives	4299	5980	71.9	fast growthfor slaughter	2272	5985	38.0
Overusing antibiotics	2987	5987	49.9	high profits	1340	5985	22.4

Table 4 shows that quite a number of people make negative comments on factory rearing of pigs and fowls–71.9% of respondents are worried about the overuse of additives; 49.9% are concerned about the overuse of antibiotics and 48.3% complain about “bad taste”. In positive comments, 38.0% select “fast growth for slaughter”; 33.3% choose “high productivity” and 22.4% think it has a satisfying commercial return. Generally, on the topic of factory rearing, negative comments by the public outnumber positive ones.

Given the fact that there were more respondents express negative views, are people willing to pay more for animal-friendly production with a higher cost? Take an instance of pork purchasing, among the 5,974 respondents, 564 of them (9.4%) are “gladly willing to” spend more; 2,693 of them (45.1%) are willing just “to some degree”; 2,029 people (34.0%) show reluctance; and 688 of them (11.5%) say no to it. Shown in the above data, a little more than half of the public are willing to pay more for pork reaching the standards of animal welfare.

### The Public Attitudes to Animal Welfare Legislation

Generally, there is a global agreement that animal welfare is not only a moral issue but also a legal one. So far, more than one hundred states have established laws on animal welfare, but China is not one of them. Do Chinese also need such laws? Among 5,772 respondents, 4,712 of them think it is necessary; whereas 1,060 of them don’t think so, with the proportion being 81.6% and 18.4% respectively. Judging from this, the necessity of establishing animal welfare laws is widely recognized by the public in China.

Nevertheless, when it comes to specific animals and certain human behavior, what will happen to public opinion? To the question “Do you agree on establishing mandatory laws of animal welfare to compel producers to provide better living conditions for farm animals such as pigs and fowls to help them grow and survive?”, 1,249 people, 20.8% of the 5,996 respondents, express complete approval; 2,699 of them (45%) approve “to some degree”; 188 of them (3.1%) disapprove completely; 856 of them (14.4%) have never thought about it; and the answer from 1,004 people (16.7%) is “not completely approving”. Compared to previous data, the proportion of people who approve (including both “completely approving” and “to some degree approving”) has a drop of 16 percent from 81.6% to 65.99%, while the proportion of those who disapprove (including “not completely approving” and “completely disapproving”) has slightly risen to 19.8% from 18.4%, though there are some respondents “never having thought about it”.

### Analysis of Satisfaction on the Market Supply of Pigs and Fowls

As for the situation of pork supply in China’s market, among the 5,976 respondents, 1,176 of them (19.7%) feel “satisfactory”; 1,967 of them (32.9%) choose “to some degree satisfactory”; 2,056 of them (34.4%) are “not very satisfactory”; and 777 people (12.9%) regard it as unsatisfactory. In general, more people, 52.6% of the whole sample quantity, feel satisfied (their answers include “satisfactory” or “to some degree satisfactory”). Concerning the situation of egg supply in China’ market, among the 5,967 respondents, 1,412 of them (23.7%) feel “satisfactory” and 1,952 people (32.7%) make the choice of “to some degree satisfactory”; while 2,033 of them (34.1%) feel “not very satisfactory” and 570 of them (9.6%) are “unsatisfactory”. In general, there are more people, 56.4% of the whole sample quantity, who express satisfaction (including “satisfactory” or “to some degree satisfactory”).

But what lead to people’s dissatisfaction on the supply of pork and egg? Table 5 probes into this question, showing that pork receives lower level of satisfaction than egg in the market.

**Table 5:** Chinese Public’s Satisfaction Degrees on Market Supply of Pork and Egg.

	The first three mostunsatisfactory items ofpork supply in China’s market						The first three mostunsatisfactory items of eggsupply in China’s market					
	Themost unsatisfactory		The second most unsatisfactory		The third most unsatisfactory		Themost unsatisfactory		Thesecond most unsatisfactory		The thirdmost unsatisfactory	
	n	%	n	%	n	%	n	%	n	%	n	%
Deficiencyof supply	203	4.9	126	3.7	138	5.1	237	6.1	140	4.7	153	6.6
Higherprice	2049	49.7	610	18.1	324	12.0	1725	44.3	538	18.0	316	13.6
Taste worse than before	662	16.1	990	29.4	475	17.6	931	23.9	1037	34.7	435	18.7
Uncertainty of food safety	974	23.6	1071	31.8	693	25.7	811	20.8	868	29.0	639	27.4
Weak market supervision	189	4.6	514	15.3	772	28.6	141	3.6	355	11.9	561	24.1
Others	47	1.1	51	1.5	292	10.8	52	1.3	50	1.7	228	9.8
N	4124	100.0	3362	100.0	2695	100.0	3898	100.0	2988	100.0	2332	100.0
Proportion	68.7		56.0		44.9		64.9		49.8		38.8	

Then the researchers measure the levels of satisfaction on pork and egg respectively. In Table 5, under the category of “the most unsatisfactory” of pork, the reason chosen by the largest number of respondents (almost half) is “higher price”, followed by “uncertainty of food safety”, then “taste worse than before”. Less than 5 percent are dissatisfied because of “deficiency of supply” or “weak market supervision”. While for egg, high price leads to the greatest level of dissatisfaction, though less than pork. The factors leading to discontentment that are ranked second and third are “uncertainty of food safety” and “taste worse than before”. Just a very small percent are disappointed by “deficiency of supply” and “weak market supervision”. Obviously, when people purchase pork and egg, the top three main reasons for dissatisfaction are price, food safety and taste.

## Factors that May Influence Public Attitudes to Animal Welfare

As this survey is conducted among citizens nationwide, it should be noted that people from different regions tend to take different attitudes on the same subject due to the gap of economic and social development. In order to obtain a more objective understanding of the influence of different factors on people’s attitudes to animal welfare, individual characteristics, including gender, age, education, career, income, birthplace and working place which are related to social attitude to animal welfare, should be taken into consideration. Table 6 shows the results of relevant data analysis.

**Table 6:** The Logistic Regression Model I of China’s Public Attitudes to Animal Welfare and its Influential Factors.

Independent Variables		V1	V2	V3	V4	V5	V6	V7	V8
Gender	B	–0.091	–0.265**	–0.235**	0.231**	0.092	0.280**	0.123	–0.091
	S.E	0.07	0.074	0.077	0.068	0.067	0.075	0.086	0.066
	Exp (B)	0.913	0.767	0.791	1.259	1.097	1.323	1.131	0.913
Age sector	B	–0.311**	0.292**	0.215**	–0.343**	0.024	0.095	–0.06	0.083
	S.E	0.074	0.073	0.076	0.072	0.069	0.077	0.087	0.068
	Exp (B)	0.733	1.34	1.24	0.712	1.024	1.1	0.942	1.087
Birthplace (urban or rural)	B	–0.006	0.312**	0.038	–0.042	–0.211*	–0.311**	–0.223*	–0.279**
	S.E	0.088	0.094	0.1	0.087	0.085	0.093	0.109	0.084
	Exp (B)	0.994	1.366	1.039	0.959	0.81	0.733	0.9	0.756
Birthplace (eastern, central or western)	B	0.147*	0.028	0.229**	0.078	–0.04	–0.104	–0.167	–0.092
	S.E	0.072	0.081	0.083	0.072	0.072	0.079	0.092	0.07
	Exp (B)	1.159	1.028	1.257	1.082	0.961	0.902	0.846	0.912
Education	B	0.368**	–0.248**	–0.229**	0.08	0.111*	0.17	0.242**	0.004
	S.E	0.055	0.053	0.055	0.052	0.05	0.055	0.061	0.05
	Exp (B)	1.445	0.781	0.742	1.083	1.118	1.186	1.274	1.004
Career category	B	–0.093	–0.179**	–0.134*	0.115*	0.178*	0.143*	0.232**	0.031
	S.E	0.061	0.061	0.064	0.059	0.058	0.064	0.074	0.056
	Exp (B)	0.912	0.836	0.875	1.122	1.195	1.154	1.262	1.031
Working place (urban or rural)	B	–0.141	–0.028	0.053	–0.015	0.231*	0.393**	0.285*	0.072
	S.E	0.117	0.116	0.123	0.113	0.11	0.12	0.136	0.103
	Exp (B)	0.869	0.973	1.054	0.985	1.26	1.482	1.33	1.075
Working place (eastern, centralor western)	B	–0.227**	–0.041	–0.114	–0.065	–0.03	0.07	0.206*	–0.076
	S.E	0.075	0.083	0.084	0.074	0.073	0.081	0.094	0.072
	Exp (B)	0.797	0.96	0.892	0.937	0.97	1.072	1.229	0.927
Annual Household income	B	0.11	0.041	0.076	0.018	0.319**	0.100*	0.113	0.092*
	S.E	0.043	0.047	0.049	0.043	0.045	0.05	0.059	0.042
	Exp (B)	1.116	1.042	1.079	1.018	1.375	1.105	1.12	1.096
Effective samplequantity		3755	3746	3746	3605	3750	3766	3673	3753
Constant		–0.827	–0.102	–0.214	–0.662	–1.1	–0.563	–0.191	0.49
Likelihood logarithm		4751.473	4389.244	4119.78	4838.019	5020.266	4317.292	3452.232	5151.484
chi-squarevalue		185.055	211.159	158.935	94.664	135.59	76.172	102.478	39.858

Note: **P<0.01 and *P<0.05 were through dual-trail verification.

In Table 6, all the dependent variables are forced into model through the approach of “Enter” statistics. It reflects influence on each dependent variable from each independent variable. For instance, the four independent variables of education, birthplace (divided by eastern, central and western), age and working place (divided by eastern, central and western) have notable impact on whether the respondents are aware of concept of animal welfare (V1) or not. Accordingly, the respective influential factors of the dependent variables V1–V9 can be deduced.

It is necessary to carry out further analysis because there are other determining factors, such as general approach and mechanism of the spread of new concepts or ideas, level of acceptance of legislation by different social groups, and degree of sensibility to price. Hence, manual screening is adopted to remove the variables which do not markedly affect the dependent variables in Table 6. The findings are reported in Table 7.

**Table 7:** The Logistic Regression Model II of China’s Public Attitudes to Animal Welfare and its Influential Factors.

Independent variables		V1	V2	V3	V4	V5	V6	V7	V8	V9
Gender	B		–0.237**	–0.248**	0.200**		0.227**			–0.241**
	S.E		0.069	0.072	0.060		0.073			0.060
	Exp (B)		0.789	0.780	1.222		1.255			0.786
Age sector	B	–0.270**	0.339**	0.225**	–0.425**					
	S.E	0.066	0.069	0.071	0.060					
	Exp (B)	0.763	1.403	1.253	0.654					
Birthplace (urban or rural)	B		0.272**			–0.202*	–0.323**	–0.185	–0.151**	
	S.E		0.073			0.081	0.090	0.103	0.058	
	Exp (B)		1.313			0.817	0.724	0.831	0.859	
Birthplace (eastern, central or western)	B	0.044		0.090						
	S.E	0.064		0.047						
	Exp (B)	1.045		1.094						
Education	B	0.349**	–0.239**	–0.282**		0.096*		0.252**		
	S.E	0.040	0.049	0.050		0.046		0.055		
	Exp (B)	1.418	0.788	0.754		1.100		1.287		
Career category	B		–0.211**	–0.156**	0.145**	0.213**	0.234**	0.231**		0.087*
	S.E		0.057	0.059	0.041	0.054	0.051	0.068		0.038
	Exp (B)		0.810	0.856	1.156	1.238	1.264	1.260		1.091
Working place (urban or rural)	B					0.231*	–0.365**	0.182		
	S.E					0.103	0.115	0.127		
	Exp (B)					1.260	1.441	1.200		
Working place (eastern, central or western)	B	–0.152*						0.014		
	S.E	0.067						0.056		
	Exp (B)	0.859						1.015		
Annual family income	B					0.308**	0.130**		0.128**	0.130**
	S.E					0.042	0.047		0.035	0.037
	Exp (B)					1.360	1.139		1.137	1.139
Effective sample quantity		4588	4357	4360	4673	4094	3963	4212	5103	4542
Constant		–1.148	–0.108	0.080	–0.339	–1.093	–0.098	0.146	0.130	0.194
Likelihood logarithm		5845.928	5025.725	4744.709	6288.666	5493.409	4550.549	3937.298	7033.893	6187.647
Chi-square value		183.860	269.779	180.704	110.939	139.737	62.362	99.193	24.664	36.675

Note: **P<0.01 and *P<0.05 were through dual-trail verification.

In Table 7, variables of birthplace (divided as eastern, central and western) no more markedly affect V1 and V3; variables of birthplace (divided as urban and rural) and working place (divided as urban and rural, and divided as eastern, central and western) no longer have significant influence on V7.

According to Table 7, the citizens with higher educational backgrounds, lower age, and working in eastern regions are more likely to be aware of animal welfare (V1); those at older ages, born in rural areas, with lower educational backgrounds, male citizens and those engaging in relatively simple career (such as farmers) are more inclined to support the use of cement flooring for pig rearing (V2); those with lower educational backgrounds, male citizens, those at older age and engaging in relatively simple career are more likely to consider killing fowls near their cage (V3) as appropriate; citizens at lower age, female citizens and those engaging in relatively complicated career (such as in government and NGO) are more likely to support factory rearing (V4); those with higher annual household income, working in rural areas, engaging in relatively complicated career, born in urban regions and those of higher educational backgrounds are more willing to pay more for the more expensive animal products living with better animal welfare (V5); those born or working in urban regions, engaging in relatively complicated career, female citizens and those generating higher annual household income are more likely to agree on making mandatory laws of animal welfare (V6); those having higher educational backgrounds, female citizens and engaging in relatively complicated career are more likely to accept the idea of learning from abroad to establish laws of animal welfare (V7); those born in urban regions and those with higher annual household income are more likely to feel satisfied with the pork supply in China (V8); female citizens, those with higher annual household income and those engaging in relatively complicated career are more inclined to be satisfied with the egg supply in China (V9).

## Discussion

The survey conducted by the researchers reveals that only about one third of Chinese public have ever heard of animal welfare. In other words, most Chinese have never heard of it. Moreover, considering relatively high educational level and young age of the respondents, it is possible that the real proportion of Chinese who have ever heard of animal welfare would be even lower. Consequently, though the past decade saw the spread of the concept of animal welfare after its introduction to China from the west, it has not been truly popularized in China partly due to the lack of introduction via mainstream media. Public awareness for animal welfare, on both legislation and farming system, still need to be enhanced thus there is a long way to go for China to promote the concept of animal welfare.

While most Chinese have never heard of animal welfare, this does not mean that Chinese people do not care about the well-being of animals. As a developing country, China needs to adopt factory rearing so as to meet the growing demand for products of livestock and fowls. The government and media should carry on massive and extensive campaign to improve public awareness on such issue. However, the survey has found that 44% of respondents make negative comments on current factory rearing; 23.8% think it limits the freedom of pigs and domestic fowls; 20.2% regard it as a cruel way of production of pigs and fowls. The opposition to current rearing system reflected from this survey matches, to some extent, to a conclusion in Welfare Quality Projects that, significantly, consumers believe that intensive systems are unnatural and, therefore, unsafe. [6] On several detailed welfare aspects, Chinese public shows a high level of approval. For instance, when asked about the statement “Though pigs naturally like to nose the earth, most of the piggeries use cement floor.”, 69.7% of respondents consider it to be not very appropriate or extremely inappropriate. When asked about whether it is appropriate to kill fowls near their cages when they are sold in market, as high as 74.3% of respondents oppose it by choosing “not very appropriate or extremely appropriate”. For Chinese people, even though they have never heard of the concept of animal welfare, they are very likely caring for animals in mind, influenced by traditional ethics in such as Confucianism, Taoism, and Buddhism. Therefore, Chinese cultural legacy might make contribution to the promotion of welfare farming as a psychological driving force.

In this study, among the answers to the question “Do you agree on establishing mandatory laws for animal welfare to compel producers to provide better living conditions for farm animals such as pigs and fowls to help them grow and survive?”, 65.8% of respondents completely or partly agree, and 19.8% completely disapprove or not very much approve of it, with 14.4% “never having thought about it”. The data shows that there do exist positive social background for promoting animal welfare legislation in China. However, this background may vary in different social stratifications. Based on data in Table 6 and Table 7, the idea of animal welfare is more likely to be held by people with relatively sophisticated professions (related to higher educational background and career prestige), higher educational background (related to better jobs with higher pay), people working in eastern regions (where there are more developed social and economic situation and the related better living conditions), and people at a younger age (more open and sensitive to new ideas). In addition, the young and the highly educated tend to accept more easily the concept of animal welfare. These findings match, to some extent, a survey conducted by Welfare Quality Project on the points of view of citizens in several European countries which showed that “generally everybody cares about animal welfare, both in general and in relation to food production” [7].

With the application of factory farming system, improved living conditions of pigs means not only improving the quality of pork, but also resulting in higher cost of production. Among all the respondents, 9.4% of them are quite willing to spend more; 45.1% are “to some degree willing to do so”; 34.0% show reluctance; and 11.5% are unwilling to do so. These findings imply an existing advantage for China to improve animal welfare. Anyway, it is not wise to be too optimistic because people’s support is based on the condition that the meat quality will be improved and 45% of the respondents are completely or partly not willing to pay more. Therefore, it is necessary to realize that welfare rearing in China will still be hindered by many societal barriers.

However, what consumers think do not always influence what they have being doing. A majority of Norwegians (57%) believe that animal welfare should be considered to a greater extent, while 38% are content with the situation today. Although many seem to worry about animal welfare in food production, it doesn’t seem to influence their consumption of meat and fish to a great extent. [8] Therefore, instead of the being dependent on voluntary behavior change from consumers, animal welfare legislation could be a necessary way to get a quick and obvious result. Nevertheless, while there has been, to some extent, social supports for animal welfare improvement in which the most practical way might be the animal welfare legislation, it may take long time to achieve this tough object. A comprehensive time schedules supplemented to rigid laws may have to be adopted. Bennett and Blaney empirically showed that the vast majority of UK respondents are concerned about animal welfare and supported proposed legislation to phase out the use of battery cages for egg production within the EU. [9] EU legislation allows for transitional periods of several years in order to facilitate the implementation of structural changes in certain farming systems; however, this approach has not always led to timely conversion. Indeed cultural appreciation of animal welfare aspects plays a fundamental role in enhancing the respect of both the spirit and the actual stipulations of the legislation [10].

In conclusion, it can be found that the majority of the public in China take a stand of weak anthropocentrism–their support for improving rearing conditions of pigs and fowls stems largely from their hope for better food quality and safety of animal products. This standpoint is different from the gist of such animal welfare laws as “Martin’s Act” advocating people to respect animal lives, protect animal rights, and not abuse animals, which should be considered in the promotion of animal welfare in China.
